# Self-appreciation is not enough: exercise identity mediates body appreciation and physical activity and the role of perceived stress

**DOI:** 10.3389/fpsyg.2024.1377772

**Published:** 2024-09-10

**Authors:** Linyu Shi, Lixia Jiang, Song Zhou, Wenbo Zhou, Huaqi Yang

**Affiliations:** ^1^Department of Psychiatry, Beijing Children’s Hospital, Capital Medical University, National Center for Children’s Health, Beijing, China; ^2^School of Psychology, Fujian Normal University, Fuzhou, China; ^3^China Basketball College, Beijing Sport University, Beijing, China

**Keywords:** body appreciation, exercise identity, perceived stress, physical activity, self-determination theory

## Abstract

**Introduction:**

This study explores the relationship between body appreciation and physical activity, focusing on the mediating role of exercise identity and the moderating effect of perceived stress. While individuals with positive body image are generally thought to engage in proactive physical activity, it remains unclear whether this positive attitude necessarily promotes exercise.

**Methods:**

We conducted a short-term longitudinal survey, recruiting 345 college students 28 (100 females, 245 males; *M*_age_ = 22.94, SD = 5.99) who completed questionnaires at two-week intervals for a total of three times within four weeks. Body appreciation, exercise identity, perceived stress, and physical activity were measured for the participants separately.

**Results:**

The results demonstrated that body appreciation positively predicted physical activity, exercise identity partially mediated the positive effect of body appreciation on physical activity, and perceived stress played a moderating role in body appreciation and exercise identity.

**Discussion:**

These results highlight the significant role of body appreciation in influencing physical activity through exercise identity, with perceived stress further moderating this relationship. The study underscores the importance of promoting body appreciation and regulating stress to enhance physical activity engagement among college students.

## Introduction

In recent years, the physical activity level of college students has decreased. A survey result showed that 22% of people aged 18–29 exercise less than once per week, and only 1.4% work out five or more times per week (Billitz, 2023). Compared to high-intensity physical activity, low-intensity and moderate-intensity physical activity is more likely to induce depression and anxiety (Li et al., 2022), potentially leading to further reductions in physical activity levels and exacerbating mental health issues. Therefore, it’s of great importance to explore the driving mechanisms of physical activity among college students and motivate them to engage in physical activity.

Physical activity is defined as any bodily movement produced by skeletal muscle that results in the consumption of energy (Caspersen et al., 1985). It’s believed to be the most common way to maintain physical and mental health, which enhances immunity (Lin et al., 2022), prevents disease (Ginis et al., 2017), as well as reduces stress (Cao et al., 2021), and increases self-confidence (Bal et al., 2020). College students are at a critical period in their lives, and improved physical activity is beneficial for academic engagement (Han and Ju, 2022) and future sustainable development. Thus, guiding them to increase their participation in physical activity is an important goal. At present, the existing reasons affecting undergraduate students’ physical activity are diverse, including individual, cognitive, social, and environmental factors (Choi et al., 2017). In this study, we primarily focus on the internal factor, especially the effect of motivation on behavior. A study based on self-determination theory (SDT) suggests that autonomous rather than controlled motivation leads to sustained physical activity (Ntoumanis et al., 2021). However, how motivation contributes to physical activity remains unknown. Therefore, to explain the internal mechanism of college students’ physical activity, it is necessary to further elucidate the specific pathway of motivation to behavior.

### Body appreciation and physical activity

Based on self-determination theory, Cox et al. (2019) suggested that positive body image may be linked more tightly to autonomous forms of motivation. Body appreciation is the hallmark of positive body imagery, which includes accepting the body, maintaining a good attitude toward the body, and respecting the body by taking care of its needs and engaging in healthy behaviors, while rejecting the media-promoted ideals of appearance as the only form of beauty (Avalos et al., 2005). Cox et al. (2019) found that body appreciation is one of the internal factors that promote healthy behaviors among younger adults. Individuals who appreciate their bodies are more likely to engage in health-promoting behaviors such as physical activity and enjoy better sleep quality (Andrew et al., 2016); they tend to avoid behaviors that negatively affect physical and mental health, like excessive exercise (Vani et al., 2021). Conversely, a lack of body appreciation is related to poor health practices and experiences, including reduced physical activity and poorer sleep quality (Aquil et al., 2021). The physical self-concept is an important area of self-concept. Previous studies have found that body self-concept is a determinant of an individual’s physical activity and can be used to explain changes in physical activity (Marsh et al., 2006). Individuals’ perceptions and thoughts about their bodies may increase or decrease their participation in physical activity. For example, studies of adolescent girls have found that high levels of body appreciation predicted increased physical activity a year later (Andrew et al., 2016), and girls would avoid exercise because of their appearance concerns (Moreno-Murcia et al., 2011). Therefore, we proposed that body appreciation positively predicts the extent of physical activity.

### Mediating effect of exercise identity

Exercise identity, defined as the extent to which an individual identifies himself or herself as an exerciser, represents an emphasis on one’s previous exercise behavior and guide their future exercise behavior (Anderson and Cychosz, 1994, 1995). Schwartz et al. (2011) employed cross-lagged Models to find a reciprocal relationship between self-concept clarity and personal identity. In other words, self-concept clarity can predict future personal identity and personal identity can predict future self-concept clarity. High levels of body appreciation, i.e., clear body self-concept, may influence adherence to an exerciser’s identity. People’s positive perceptions of their bodies may influence their purpose for engaging in physical activity. Systematic review and meta-analysis found strong correlations between physical activity and perceived competence and perceived fitness in adolescents, including those from diverse cultural backgrounds such as Asia, compared to perceived appearance (Babic et al., 2014), this may make them more likely to identify themselves as an exerciser. In addition, an exercise-based meta-analysis of 121 studies found that exercisers reported higher positive body image compared to non-exercisers (Hausenblas and Fallon, 2006). Therefore, we speculated that body appreciation may have an effect on exercise identity for college students. Role identity theory posited a close relationship between role-based identity and behavior, individuals align their actions with their role identities to maintain consistence (Stryker and Serpe, 1982). Role identity can moderate the influence of motivation on physical activity, thereby enhancing physical activity (Amireault and Huffman, 2024). Exercise identity reflects an individual’s commitment to the exerciser role, motivating regular physical activity to reaffirm this identity (Anderson and Cychosz, 1995). Studies revealed that people with a high level of exercise identity were more physically active and had higher levels of frequency, intensity, and duration of physical activity participation than people with a low level of exercise identity (Anderson and Cychosz, 1994; de Bruijn and van den Putte, 2012). In addition, a recent study using cross-lagged panel modeling found that exercise identity affected future physical activity (Porter et al., 2024).

Therefore, we proposed that body appreciation may influence college students’ physical activity levels through exercise identity.

### Moderating effect of perceived stress

Perceived stress may affect exercise identity, which diminishes the positive effects of body appreciation on physical activity. Stress can be defined as a reaction state that occurs when an individual perceives a situation as threatening or challenging and they lack the resources to cope with the stress (Cohen et al., 1983). There is a complex relationship between body appreciation and perceived stress. Body appreciation can enhance self-esteem and maintain mental health (Avalos et al., 2005; Swami et al., 2024). However, persistent perceived stress may diminish these positive outcomes (Nguyen et al., 2019), and disruptive identity-related events may lead to emotional and psychological distress. When people are unable to cope with stress, they become anxious, depressed, stagnant, and have less energy and time to engage in beneficial activities, such as exercise (Stults-Kolehmainen and Sinha, 2014; Knepp et al., 2015). Stults-Kolehmainen and Sinha (2014) found that people under chronic stress were less likely to participate in physical activity compared to people with less stressful. When individuals believed that they were forced to display their body and likely to be criticized by others, they may develop negative emotions related to the exercise and weaken their motivation to exercise. A recent qualitative study showed that physical visibility in physical education classes could increase students’ perceived stress, which exacerbates their body image concerns, and further affects their participation in exercise (Åsebø et al., 2022). In addition, previous research has demonstrated that high-pressure environments or sudden stressful events could threaten an individual’s self-concept clarity and cause self-concept confusion (Ritchie et al., 2011). Individuals with impaired self-concept clarity may have difficulty maintaining their identity as exercisers and consequently reduce participation in physical activity.

Therefore, we proposed that perceived stress may moderate the effects of body appreciation on exercise identity, leading to increased or decreased levels of physical activity.

### The present study

**Figure 1:**
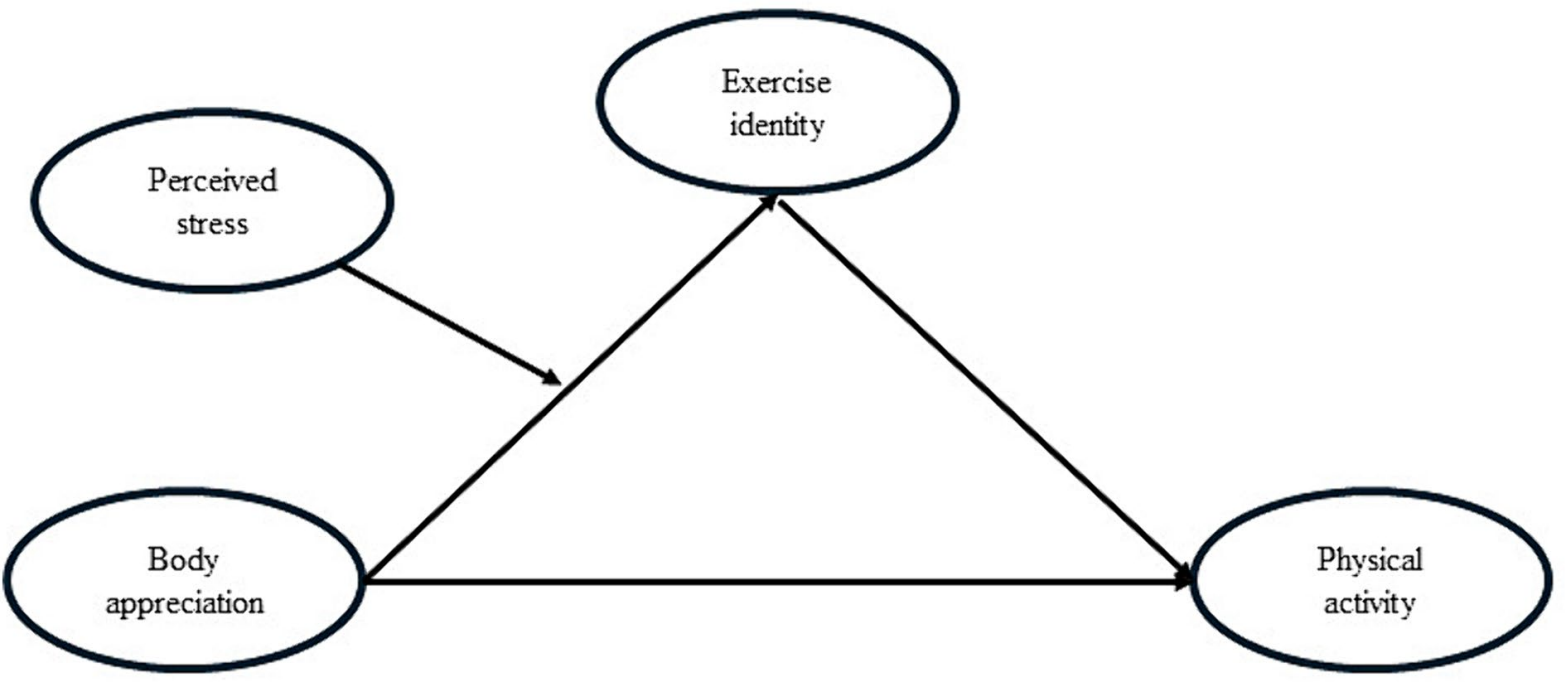
The assumed model in this study.

Previous studies suggested that body appreciation can promote physical activity. Exercise identity may mediate the relationship between body appreciation and physical activity, and perceived stress plays a moderating role in the relationship between body appreciation and exercise identity. The purpose of this study is to explore the specific mechanisms of action between body appreciation and physical activity, and to provide a theoretical reference for promoting college students’ physical activity. We proposed the following hypotheses and constructed a moderated mediation model to test the hypotheses (see Figure 1).

*H1*: Body appreciation positively predicts physical activity;

*H2*: Exercise identity mediates the effect of body appreciation on physical activity;

*H3*: Perceived stress plays a moderating role in the relationship between body appreciation and exercise identity.

## Methods

### Participants and procedures

This study used a short-term longitudinal study design. We conducted a short-term longitudinal study at a university in Beijing with a duration of 4 weeks, wherein a total of 3 tests were implemented with an interval of 2 weeks between each test. Undergraduate students who were healthy, and without disabilities were selected as participants. Three hundred and seventy-nine undergraduate students were initially recruited at the beginning of the current study (T1). Participants completed body appreciation scale, physical activity scale and provided their demographic information. Two weeks later (T2), participants completed exercise identity scale and perceived stress scale, and returned 360 questionnaires. At the ending time point of the current study (T3), 367 participants completed physical activity scale. Finally, 345 participants’ data were left for formal analysis (100 females, 245 males; $M_{\mathrm{age}}$ = 22.94, SD = 5.99). All participants provided written informed consent, and those who participated in all three surveys received 5 yuan as rewards. This study was approved by the [redacted for peer review].

### Measurements

#### Body appreciation

Body Appreciation Scale-2 used in this study was developed by Tylka and Wood-Barcalow (2015a), and the Chinese version was translated by Swami et al. (2016). The 10-item scale measured if participants appreciate their body. It includes questions like “I respect my body.” and “I feel that my body has at least some good qualities.” on a 5-point scale (1 = not at all, 5 = most of the time). The higher the score, the higher the level of body appreciation. The Cronbach’s alpha was 0.90 in this study.

#### Physical activity

Physical activity of the participants was measured with reference to the two items used in Zhou et al. (2021), which were adapted from the International Physical Activity Questionnaire (Booth, 2000), and participants reported on their degree of participation in physical activity. One of the items was “In the previous 2 weeks, how many times did you exercise on average per week?.” Participants responded on a 5-point scale (1 = none; 2 = 1–2 times; 3 = 3–4 times; 4 = 5–6 times; and 5 = 7 times or above). The other item was “In the previous 2 weeks, how often did you engage in *physical activities?.” The items were rated on a 4-point scale (1 = never; 2 = rarely; 3 = sometimes; 4 = often; 5 = very often). A higher score indicated a higher level of physical activity. The Cronbach’s alpha was 0.85 in this study.

#### Exercise identity

Exercise identity was measured using the Exercise Identity Scale (EIS; Anderson and Cychosz, 1994), and its reliability and validity in the Chinese college students have been well documented (Meng-long et al., 2019). The scale consists of 9 items to assess the extent to which an individual is an exerciser, three of which measured role identity (e.g., “I consider myself to be an exerciser.”) and six items measured exercise beliefs (e.g., “Most of the time I spend thinking about the sport”). The items were rated on a 7-point scale (1 = Completely disagree, 7 = Completely agree). Higher scores reflect higher exercise identity. In this study, the Cronbach’s alpha was 0.96.

#### Perceived stress

Perceived stress was measured by two items used in the Zhou et al. (2021), with items derived from the Perceived Stress Scale developed by Cohen et al. (1983). The scale was used to reveal the degree of perceived stress in the lives of college students. The items include “In the last month, how often have you felt that you were unable to control the important things in your life?” and “In the last month, how often have you felt difficulties were piling up so high that you could not overcome them?.” Each item is rated on a scale of 1 (never) to 5 (very often). A higher score indicated a higher level of perceived stress. The Cronbach’s alpha was 0.91 in this study.

#### Sociodemographic variables

Participants’ age, gender, yearly family income, and subjective social status (from 1 = the lowest to 10 = the highest) were measured as control variables.

### Data analysis

Firstly, we excluded invalid samples who did not complete the three tests. We then generated descriptive statistics and correlations for all study variables.

For the main analysis, we used measurement data at different sampling times to construct the assumed model. Specifically, body appreciation at T1, exercise identity and perceived stress at T2, and physical activity at T3 were included in analysis. Besides, sociodemographic variables and physical activity at T1 were treated as control variables. Based on the research method proposed by Zhonglin and Baojuan (2014), this study standardized all the variables, applied Mplus 8.3, and used Bootstrap method to extract 5,000 samples to estimate the 95% confidence intervals of each effect, and examined the hypothesized moderated mediation effect model. Unstandardized regression coefficient of the regressive path (*b*), Standard Error (*SE*), 95% confidence interval (CI) of regression coefficient, and *p*-value were reported, with significant interactions (*p* < 0.05) probed via simple slopes analysis at high (+1SD) and low (−1SD) levels of the moderator.

Finally, we used network analysis to assess the associations between body appreciation, exercise identity, perceived stress, and physical activity at both factor and item levels, and JASP 0.18.3.0 to validate the rationality of the structural equation modelling and to identify the most influential nodes at low-stress levels. According to Epskamp and Fried (2018), we applied gLASSO and EBIC with a tuning parameter of 0.5. The network system includes nodes and edges. The centrality index of the nodes in the network represents the study variables, calculated as betweenness, closeness, and strength of the nodes. The connection between nodes is described by edges, where thicker edges indicate stronger correlations and thinner edges indicate weaker correlations.

## Results

We first performed a correlation analysis, which provided preliminary support for the subsequent analysis. The correlation matrix of body appreciation, exercise identity, perceived stress, and physical activity used to construct the assumed model was presented in Table 1.

**Table 1:** The correlation matrix of body appreciation, exercise identity, perceived stress, and physical activity.

Factor	*M*	*SD*	1	2	3	4
1. BA	3.739	0.650	–			
2. EI	5.131	1.217	0.387^***^	–		
3. PS	2.651	0.968	−0.191^***^	−0.160^**^	–	
4. PA	2.851	1.011	0.267^***^	0.471^***^	−0.131^**^	–

BA, body appreciation; EI, exercise identity; PS, perceived stress; PA, physical activity.^***^p < 0.001, ^**^p < 0.01, ^*^p < 0.05.

### Body appreciation and physical activity

We first examined the predictive effect of body appreciation on physical activity among college students. The results showed that there were significant effects for gender (*b* = −0.405, SE = 0.103, 95%CI = [−0.611, −0.207], *p* < 0.001) and baseline physical activity (*b* = 0.521, SE = 0.054, 95%CI = [0.414, 0.624], *p* < 0.001), and no significant effect for age (*b* = 0.003, SE = 0.011, 95%CI = [−0.02, 0.031], *p* = 0.793), family yearly income (*b* = −0.001, SE = 0.028, 95%CI = [−0.054, 0.053], *p* = 0.984) and self-reported social hierarchy (*b* = 0.042, SE = 0.026, 95%CI = [−0.010, 0.093], *p* = 0.102). After controlling those variables, we found that body appreciation was a significantly positive predictor of physical activity (*b* = 0.161, SE = 0.043, 95%CI = [0.078, 0.248], *p* < 0.001). Thus, Hypothesis 1 was verified.

### Mediating effect of exercise identity

Then, we added exercise identity as a mediated variable to conduct a mediation model (see Table 2). The model results showed that both body appreciation (*b* = 0.109, *SE* = 0.048, 95%CI = [0.018, 0.205], *p* = 0.023) and exercise identity (*b* = 0.178, *SE* = 0.059, 95%CI = [0.059, 0.292], *p* = 0.003) positively predicted physical activity, and body appreciation was a significantly positive predictor of exercise identity (*b* = 0.387, *SE* = 0.051, 95%CI = [0.285, 0.487], *p* < 0.001). It indicated that exercise identity plays a partial mediating role in the relationship between body appreciation and physical activity, accounting for 38.55% of the total effect (indirect effect = 0.069, *SE* = 0.026, 95%CI = [0.021, 0.123], *p* = 0.008). Thus, Hypothesis 2 was verified.

**Table 2:** The mediating effect of exercise identity between body appreciation and physical activity.

Outcome variable	Predictor variable	*R^2^*	*b*	*SE*		LLCI	ULCI
PA	BA	0.351	0.109^*^	0.048		0.018	0.205
	EI		0.178^**^	0.059		0.059	0.292
EI	BA	0.151	0.387^***^	0.051		0.285	0.487

Controlling for age, gender, yearly family income, subjective social status, and baseline physical activity.EI, exercise identity; PA, physical activity; BA, body appreciation.^***^p < 0.001, ^**^p < 0.01, ^*^p < 0.05.

### Moderating effect of perceived stress

**Figure 2:**
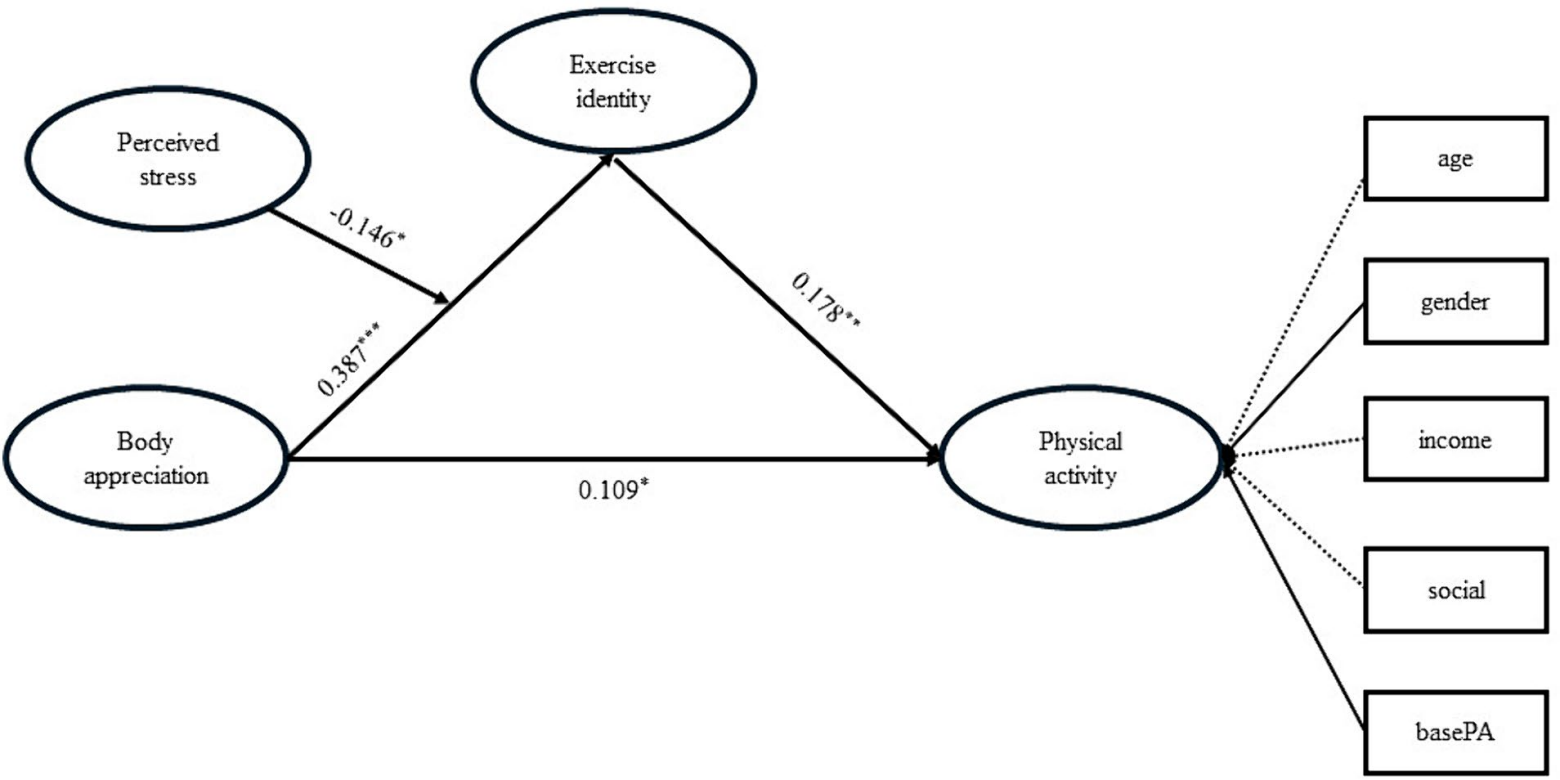
The moderation effect of perceived stress in the mediation effect of exercise identity between body appreciation and physical activity. ^***^p < 0.001, ^**^p < 0.01, ^*^p < 0.05.

**Figure 3:**
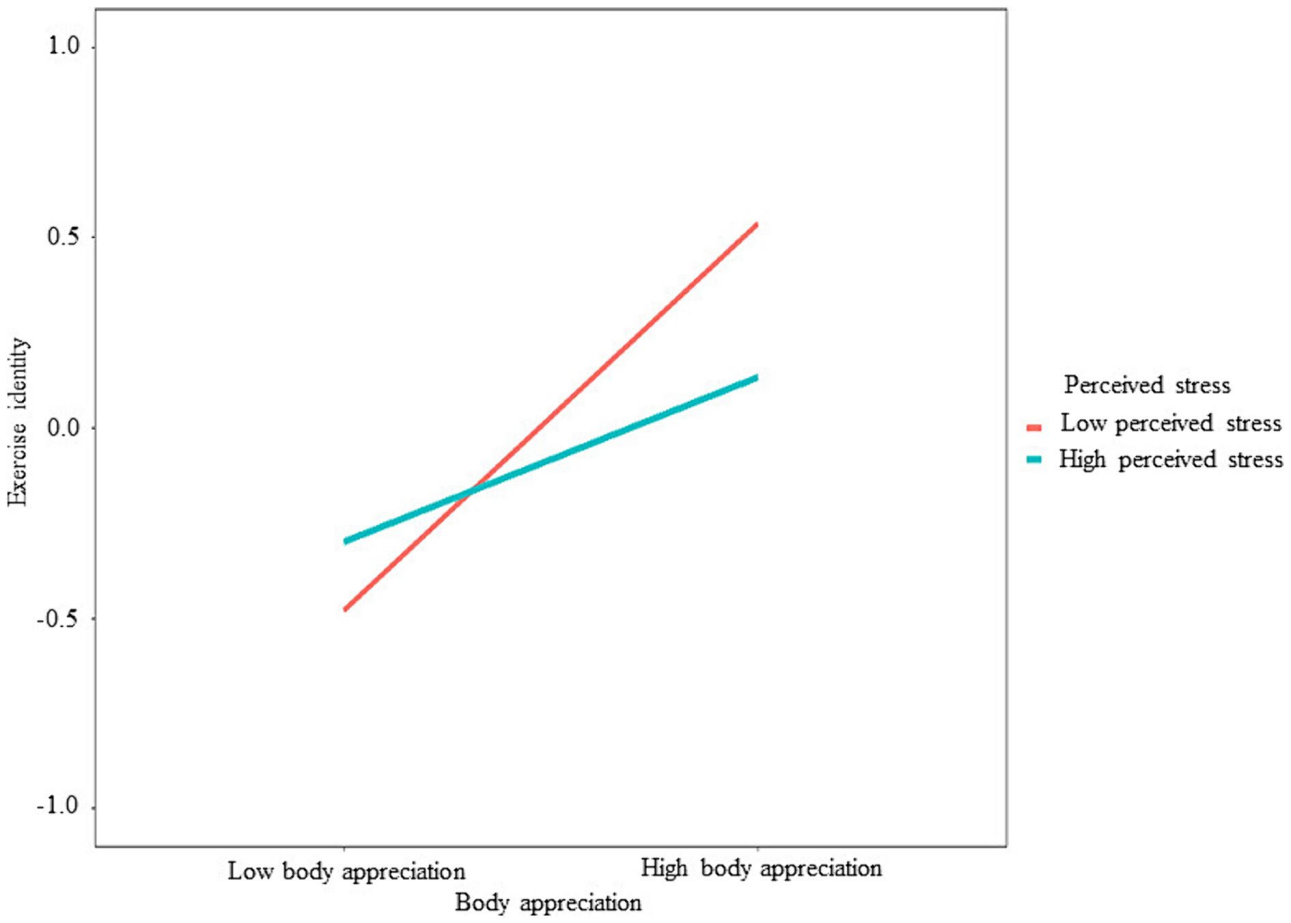
The moderating effect of perceived stress on the association between body appreciation and exercise identity.

To examine the moderating effect of perceived stress, we conduct a moderated mediation model (see Figure 2 and Table 3). Our results showed that perceived stress has a significant moderating effect on the relationship between body appreciation and exercise identity (*b* = −0.146, *SE* = 0.061, 95%CI = [−0.266, −0.030], *p* = 0.016). We then divided participants into two groups based on their scores of perceived stress, those whose stress score was one standard deviation lower than the mean were assigned to the low-stress group, and one standard deviation higher than the mean to the high-stress group. The simple slope analysis showed that the association between body appreciation and exercise identity was significant for either the high-stress group (*b* = 0.217, *SE* = 0.095, 95%CI = [0.031, 0.403], *p* = 0.022) and the low-stress group (*b* = 0.509, *SE* = 0.063, 95%CI = [0.384, 0.631], *p* < 0.001) (see Figure 3). In addition, the mediating effect of exercise identity is moderated by perceived stress, with the mediating effect being smaller in the high-stress group (*b* = 0.039, *SE* = 0.023, 95%CI = [0.028, 0.160], *p* = 0.087) than in the low-stress group (*b* = 0.091, *SE* = 0.033, 95%CI = [0.003, 0.090], *p* = 0.006). Therefore, Hypothesis 3 was verified.

**Table 3:** The moderating effect of perceived stress between body appreciation and exercise identity.

Outcome variable	Predictor variable	*R^2^*	*b*	*SE*	LLCI	ULCI
EI	BA	0.182	0.363^***^	0.053	0.258	0.468
	PS		−0.055	0.060	−0.177	0.058
	BA*PS		−0.146^*^	0.061	−0.266	−0.030
PA	BA	0.353	0.109^*^	0.048	0.018	0.205
	EI		0.178^**^	0.059	0.059	0.292

Controlling for age, gender, yearly family income, subjective social status, and baseline physical activity.EI, exercise identity; PA, physical activity; BA, body appreciation; PS, perceived stress.^***^p < 0.001, ^**^p < 0.01, ^*^p < 0.05.

### Network analysis

**Figure 4:**
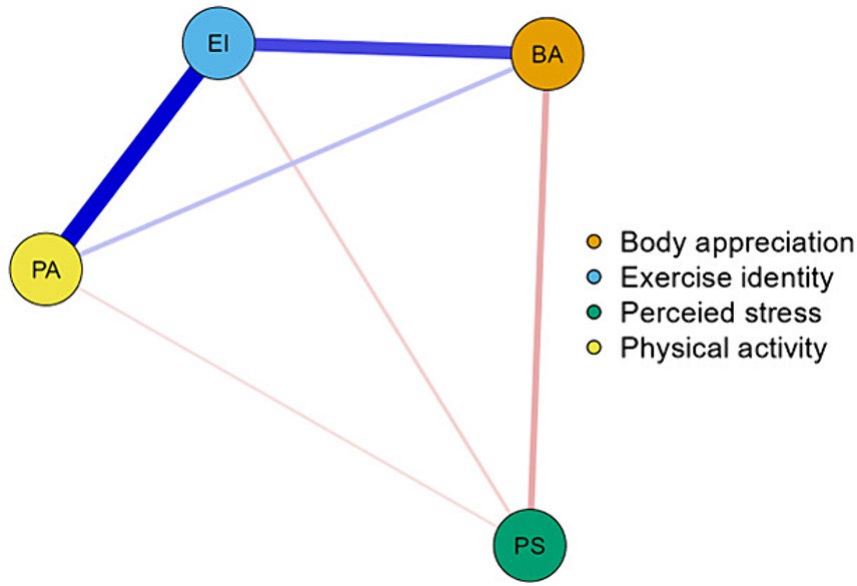
The factor-level network analysis according to the relationships between BA, EI, PS, and PA (*N* = 345). BA, body appreciation; EI1, role identity; EI2, exercise beliefs; PS, perceived stress; and PA, physical activity.

The network including body appreciation (BA), exercise identity (EI), perceived stress (PS), and physical activity (PA) for the 345 were presented in Figure 4 based on the factor-level network. There are 4 nodes and 6 non-zero edges in the network. Node EI and PA had the strongest edge intensity (*r* = 0.406). Node BA was directly associated with node EI (*r* = 0.293). In addition, the centrality measures showed that EI had the highest strength (1.149), betweenness (0.866), and closeness (0.776), indicating that EI has the most and closest connections to other nodes, and plays an important role in the network.

In order to compare the differences in network structure formed by body appreciation (BA), exercise identity (EI), and physical activity (PA) between the high-stress and low-stress groups, the network comparison test (NCT) was conducted. Firstly, the network structure invariance test showed no significant difference between the high-stress and low-stress groups (*p* = 0.287). Second, the overall strength invariance also showed no significant difference between the high- and low-stress groups (*p* = 0.310). Finally, the edge invariance test showed that only eight edges were significantly different between the high-stress and low-stress groups. Specifically, the two groups showed significant differences in edges between ba13 and ba16 (*p* = 0.021), between ba14 and ba16 (*p* = 0.002), between ba17 and ei23 (*p* = 0.021), between ba16 and ei24 (*p* = 0.012), between ba110 and ei27 (*p* = 0.008), between ba13 and ei18 (*p* = 0.031), between ei14 and pa31 (*p* = 0.031), and between ei22 and pa32 (*p* = 0.045).

**Figure 5:**
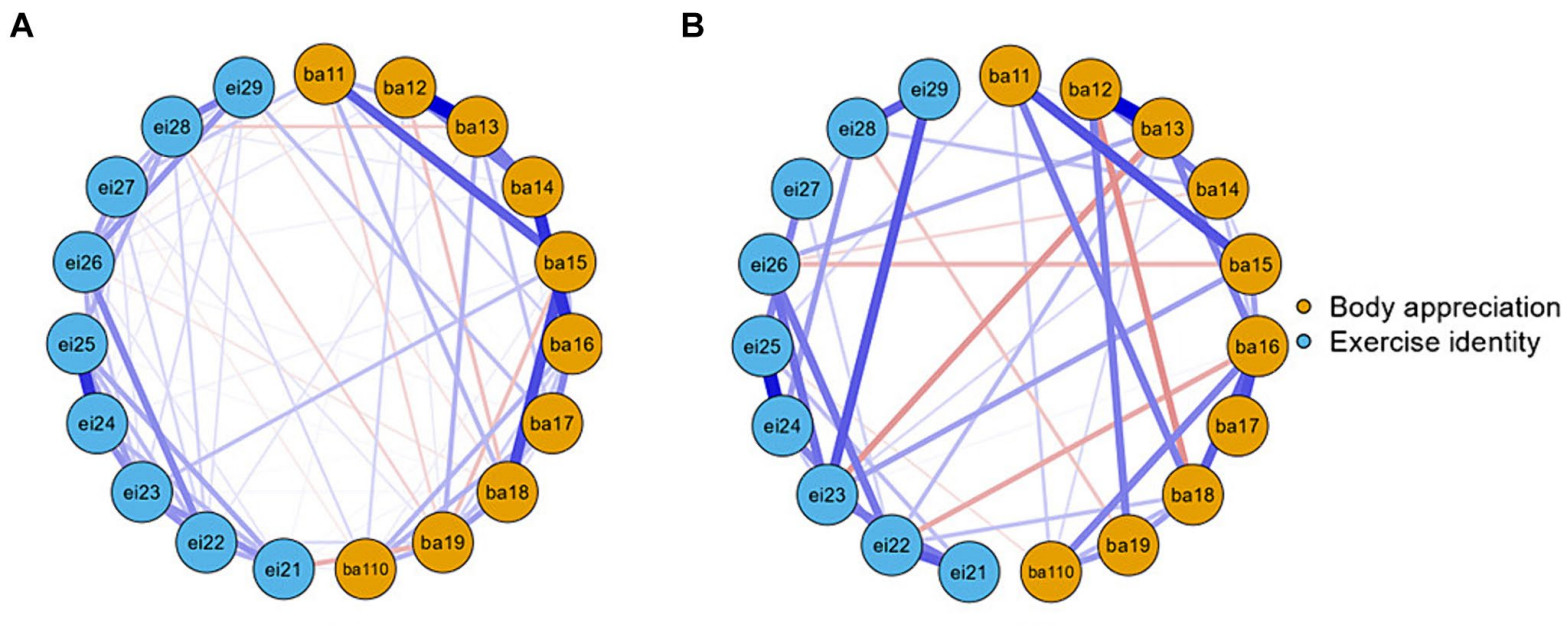
Item-level network analysis according to the relationships between BA and EI in low-stress group **(A)** and high-stress group **(B)**. BA, body appreciation; EI, exercise identity.

The network structure of the low-stress group at the item level was shown in Figure 5A, which was designed to examine the relationship between body appreciation and exercise identity as moderated by stress through which items it affects. Node ba17 (I appreciate the different and unique characteristics of my body) and node ei29 (Exercising is something I think about often) had the strongest edge intensity among participants (*r* = 0.121), node ba15 (I am attentive to my body’s needs) had a direct association with node ei23 (I have numerous goals related to exercising; *r* = 0.119), node ba11 (I respect my body) had a direct association with node ei27 (For me, being an exerciser means more than just exercising; *r* = 0.110). Besides, the centrality measures showed that ba15 had the highest betweenness (2.084), higher closeness (1.489), and strength (0.856) in the body appreciation. And ei23 had the highest betweenness (1.384), closeness (0.630), and strength (0.870) in the exercise identity. As a result, we speculated that at low-stress levels, perceived stress moderates the relationship between body appreciation and exercise identity primarily through the ba15 and ei23 pathways.

The network structure of the high-stress group at the item level was shown in Figure 5B. Node ba15 (I am attentive to my body’s needs) and node ei23 (I have numerous goals related to exercising.) had the strongest edge intensity among participants (*r* = 0.203), node ba13 (I feel that my body has at least some good qualities) had a direct association with node ei26 (Others see me as someone who exercises regularly; *r* = 0.176). Besides, the centrality measures showed that ba16 (I feel love for my body) had the highest betweenness (1.470), closeness (1.456), and strength (1.894) in the body appreciation. And ei23 had the higher betweenness (1.110), closeness (1.338), and highest strength (1.235) in the exercise identity. These results suggested that similar to low levels of perceived stress, high levels of perceived stress also moderate the relationship between body appreciation and exercise identity primarily through the ba15 and ei23 pathways.

## Discussion

While previous studies mainly focus on the relationship between body image and detrimental behaviors (Neumark-Sztainer et al., 2006), this study investigated the relationship between body appreciation and physical activity. This study aimed to explore the specific mechanisms of the relationship between body appreciation and physical activity. This study adopted the short-term longitudinal design to collect data, which reduced confounding relating to causality. The present study found that body appreciation positively predicted physical activity and that exercise identity mediated the relationship between body appreciation and physical activity. Moreover, perceived stress moderated the relationship between body appreciation and exercise identity.

### The relationship between body appreciation and physical activity

H1 was supported by our finding that body appreciation in college students can directly predict physical activity, which was consistent with previous research (Tylka and Wood-Barcalow, 2015b). This result suggested that college students who accept and cherish their bodies are more likely to engage in physical activities and other health behaviors to meet their bodily needs. Specifically, body appreciation enhances college students’ positive perceptions of their bodies, motivating them to engage in physical activities. As the Self-Determination Theory posits, autonomous motivation is a crucial factor for engaging in persistent behavior (Deci and Ryan, 2000). When college students feel satisfied and proud of their bodies, this positive perception can contribute to the development of intrinsic motivation, which in turn drives them to actively choose to participate in physical activities. Therefore, the effect of body appreciation on college students’ exercise participation is self-reinforcing, aligning with the research by Andrew et al. (2016), who found that positive body image can increase individuals’ engagement in physical activities. Furthermore, the impact of body appreciation on physical activity can also be explained by self-concept theory. A positive physical self-concept can enhance individuals’ levels of physical activity (Marsh et al., 2006). When college students have a positive view of their bodies, they are more likely to confidently engage in various physical activities to maintain and enhance their physical self-concept. Fox and Corbin, (1989) suggested that physical self-concept is a significant predictor of physical activity behavior. College students with a positive physical self-concept are motivated to engage in physical activities to sustain their positive self-perception. As a form of positive body image, body appreciation not only enhances individuals’ physical self-concept but also promotes more physical activity by boosting confidence and motivation.

### The mediating effect of exercise identity

More importantly, the study found that exercise identity mediated the relationship between body appreciation and physical activity, and H2 was supported. This result states that college students with body appreciation show higher exercise identity and physical activity. Specifically, college students’ body appreciation influences how much they identify themselves as exercisers, which in turn determines physical activity levels. Identity theory can be used to explain this finding, which posits that role identity plays a crucial role in individual behavioral choices and is a powerful predictor of behavior (Burke, 1991; Stets and Burke, 2000). When college students identify with a role, they are more likely to internalize behaviors associated with that role as part of themselves, which influences their behavioral choices. This role internalization has been linked not only to everyday decision-making, but also to individual behavioral consistency and persistence (Callero, 1985; White et al., 2008). Specifically, when college students are highly identified with the role of exercise, they are more inclined to engage in physical activity to maintain and reinforce this role identity. College students with high exercise identity are more likely to persist in exercising in the face of unfavorable circumstances because they view exercise as part of their self-concept, resulting in greater intrinsic motivation and self-confidence. On the contrary, college students with low exercise identity may lack such intrinsic motivation and are more likely to give up exercise behavior. Meanwhile, exercise identity, as a specific form of role identity, can enhance identity by boosting an individual’s self-concept and self-confidence. Body self-concept is a predictor of physical activity behavior (De la Torre Cruz et al., 2019). When college students are satisfied and proud of their bodies, they are more likely to engage in physical activity more actively by using exercise as part of their identity.

### The moderating role of perceived stress

The results of this study suggested that perceived stress moderated the effect of body appreciation on exercise identity, and H3 was supported. This result indicated that college students with body appreciation may reduce their identity with the exerciser role under the influence of stress. The presence of perceived stress makes it difficult for college students to fully internalize exercise identity as part of their self-concept in the face of body appreciation. This may be because stress can disperse the cognitive resources of students, which makes them difficult to focus their attention and energy on maintaining positive self-identity and healthy behaviors. High levels of perceived stress can lead to negative emotions such as anxiety and depression, which further reduce an individual’s motivation and confidence (Folkman, 2013), causing them to struggle to identify with the role of an exerciser. In addition, perceived stress can affect an individual’s time management and priority setting (Schneider et al., 2005). When individuals feel stressed, they may allocate more time and energy to tasks related to the source of stress, neglecting exercise and physical activity. In this situation, even if individuals have positive body appreciation, they find it hard to internalize exercise identity as part of their self-concept because exercise is no longer a priority activity. Additionally, stress can lead individuals to adopt unhealthy coping mechanisms, such as reducing physical activity and increasing unhealthy eating habits, which further weaken the formation and maintenance of exercise identity. Research has shown that individuals with high levels of stress are more likely to choose behaviors that provide short-term emotional relief rather than behaviors that offer long-term health benefits (Baum and Posluszny, 1999).

Therefore, body appreciation can promote college students’ physical activity, and their exercise identity plays a mediating role in promoting college students’ physical activity, and perceived stress can negatively affect exercise identity. The moderating mediation model constructed in this study revealed to some extent the intrinsic mechanism of body appreciation in promoting college students’ physical activity, and also brings some guiding value to the practice of improving college students’ physical activity. In order to improve physical activity, attention should be paid to raising college students’ awareness of physical activity and encouraging them to participate in various forms of sports and physical activities. Also, mental health education for students should be strengthened to help them deal with stress effectively and establish a positive attitude toward exercise, so as to improve the sense of exercise identity and promote the sustainable development of physical activity. Comprehensively using the theoretical guidance of the regulatory mediator model, targeted health promotion programs are formulated to provide scientific and comprehensive guidance for college students’ physical activity, thus further promoting the improvement of college students’ physical activity levels.

### Leveraging network analysis in the body appreciation-physical activity model

In the item-level network, there were significant positive associations between body appreciation, exercise identity, and physical activity. Also, perceived stress was negatively correlated with other variables. These results supported Hypotheses 1–3 and validated the reliability of the model. Although perceived stress differences have been reported in health status and behaviors (Roemmich et al., 2003; Mierswa and Kellmann, 2017), using the network comparison test (NCT), the relationship in the present study between body appreciation, exercise identity, and physical activity found no significant differences for perceived stress. It is often assumed that high perceived stress has more negative consequences, and the Ng and Jeffery (2003) study is consistent with this claim. Therefore, the relationship between body appreciation, exercise identity, and physical activity in the level of perceived stress requires further research to further delineate these relationships.

In the item-level network of the low-perceived stress group, ba15 and ei23 had the highest betweenness, closeness, and strength in body appreciation and exercise identification, respectively. Whereas, in the high-perceived stress group, ba15 and ei23 had the highest association in body appreciation and exercise identity, respectively. This further confirms that perceived stress has a moderating role between body appreciation and exercise identity. For the low- and high-perceived stress group, perceived stress may moderate the relationship between body appreciation and exercise identity primarily through the pathway between ba15 and ei23. Therefore, for the low- and high-perceived stress group, it is necessary to promote ba15 and ei23 to strengthen the link between body appreciation and exercise identity, thereby promoting participation in physical activity.

### Limitations and future directions

This study has several limitations that should be noticed. Though this study conducted a three-wave longitudinal survey at two-week intervals based on previous studies (Abedini et al., 2022; Liu et al., 2023), the short intervals made it difficult to detect subtle changes in the participants’ psychological characteristics during this period. Therefore, further studies may extend the sampling interval (6 months to 1 year) to obtain more effective and robust results. Additionally, this study only focuses on body appreciation (a part of positive body image), and its impact on physical activity. However, people may also develop a negative attitude toward their body image, and it may have an adverse impact on physical activity. The future studies could, respectively, explore whether and how negative body attitudes, such as body shame, influences physical activity. Thirdly, there is an unequal gender distribution, with only 27.2 percent of women in this study. Although there were no significant differences in scores between males and females on all scales in this study, the level of body appreciation tends to differ significantly between males and females. Thus, future studies could recruit populations with similar gender ratios or only focus on one gender. Fourthly, while this study investigated general perceived stress, it did not specifically address perceived stress related to body image, which could provide a more nuanced understanding of how body image issues affect overall stress levels. Future research could focus on specific perceived stress related to body image. Finally, this study only used two items to measure individual physical activity levels, which may have biased the results. Therefore, future studies will use more items to accurately measure physical activity.

## Conclusion

The current study investigated the mechanism by which body appreciation promotes physical activity. Illustrated by the results of pathway analysis, we found that exercise identity mainly mediates this effect. In addition, perceived stress moderates the mediation effect, where the positive effect is diminished as stress increases. Although previous studies have found that people who hold a positive attitude toward their body image tend to engage more in exercise, we found that body appreciation may not be the necessary factor to facilitate the level of physical activity; it relies, at least partially, on personal identity about exercisers. These findings emphasized the crucial role of role identity in helping people transform their attitudes into practice. In particular, when individuals’ identity was threatened by negative factors such as stress, they may lack the resources to persist in beneficial activities. Accordingly, we encourage college students to maintain self-appreciation while cultivating their positive attitude toward exercise to effectively promote their participation in physical activity.

## Data Availability

The datasets presented in this study can be found in online repositories. The names of the repository/repositories and accession number(s) can be found in the article/Supplementary material.

## References

[ref1] Abedini M. Jawahar I. M. Hamstra M. (2022). Does worrying about money motivate counterproductive work behavior through need satisfaction? Acad. Manag. Proc. 2022:15047. doi: 10.5465/AMBPP.2022.15047abstract

[ref2] Amireault S. Huffman M. K. (2024). Does role identity mediate the influence of motivational regulations on physical activity behavior among people 55 years or older? J. Aging Phys. Act. 32, 69–82. doi: 10.1123/japa.2022-0323 37770062

[ref3] Anderson D. F. Cychosz C. M. (1994). Development of an exercise identity scale. Percept. Mot. Skills 78, 747–751. doi: 10.1177/003151259407800313 8084685

[ref4] Anderson D. F. Cychosz C. M. (1995). Exploration of the relationship between exercise behavior and exercise identity. J. Sport Behav. 18, 159–166.

[ref5] Andrew R. Tiggemann M. Clark L. (2016). Predictors and health-related outcomes of positive body image in adolescent girls: a prospective study. Dev. Psychol. 52, 463–474. doi: 10.1037/dev0000095 26727595

[ref6] Aquil A. el O. el N. Mouallif M. Guerroumi M. Chokri A.. (2021). Body image dissatisfaction and lower self-esteem as major predictors of poor sleep quality in gynecological cancer patients after surgery: cross-sectional study. BMC Womens Health 21:229. doi: 10.1186/s12905-021-01375-5, PMID: 34082733 PMC8173912

[ref7] Åsebø E.-K. S. Løvoll H. S. Krumsvik R. J. (2022). Students’ perceptions of visibility in physical education. Eur. Phys. Educ. Rev. 28, 151–168. doi: 10.1177/1356336X211025874

[ref8] Avalos L. Tylka T. L. Wood-Barcalow N. (2005). The body appreciation scale: development and psychometric evaluation. Body Image 2, 285–297. doi: 10.1016/j.bodyim.2005.06.002 18089195

[ref9] Babic M. J. Morgan P. J. Plotnikoff R. C. Lonsdale C. White R. L. Lubans D. R. (2014). Physical activity and physical self-concept in youth: systematic review and meta-analysis. Sports Med. 44, 1589–1601. doi: 10.1007/s40279-014-0229-z 25053012

[ref10] Bal E. Sunay H. Uyar Y. Kaya B. Bianco A. (2020). The effect of regular physical activity on women’s self-confidence levels: an exploratory research. ResearchGate 3607–3512. doi: 10.19193/0393-6384_2020_6_553

[ref11] Baum A. Posluszny D. M. (1999). Health psychology: mapping biobehavioral contributions to health and illness. Annu. Rev. Psychol. 50, 137–163. doi: 10.1146/annurev.psych.50.1.137 10074676

[ref12] Billitz J. (2023). 17 college student exercise statistics (Rates & Factors). NOOB GAINS. Available at: https://www.noobgains.com/exercise-statistics-college-students/ [Accessed January 28, 2023].

[ref13] Booth M. (2000). Assessment of physical activity: an international perspective. Res. Q. Exerc. Sport 71, 114–120. doi: 10.1080/02701367.2000.11082794 25680021

[ref14] Burke P. J. (1991). Identity processes and social stress. Am. Sociol. Rev. 56, 836–849. doi: 10.2307/2096259

[ref16] Callero P. L. (1985). Role-identity salience. Soc. Psychol. Q. 48:203. doi: 10.2307/3033681

[ref17] Cao B. Zhao Y. Ren Z. McIntyre R. S. Teopiz K. M. Gao X.. (2021). Are physical activities associated with perceived stress? The evidence from the China health and nutrition survey. Front. Public Health 9:697484. doi: 10.3389/fpubh.2021.697484, PMID: 34414158 PMC8369204

[ref18] Caspersen C. J. Powell K. E. Christenson G. M. (1985). Physical activity, exercise, and physical fitness: definitions and distinctions for health-related research. Public Health Rep. 100, 126–131. 3920711 PMC1424733

[ref19] Choi J. Lee M. Lee J. Kang D. Choi J.-Y. (2017). Correlates associated with participation in physical activity among adults: a systematic review of reviews and update. BMC Public Health 17:356. doi: 10.1186/s12889-017-4255-2 28438146 PMC5404309

[ref20] Cohen S. Kamarck T. P. Mermelstein R. J. (1983). A global measure of perceived stress. J. Health Soc. Behav. 24, 385–396. 6668417

[ref21] Cox A. E. Ullrich-French S. Tylka T. L. McMahon A. K. (2019). The roles of self-compassion, body surveillance, and body appreciation in predicting intrinsic motivation for physical activity: cross-sectional associations, and prospective changes within a yoga context. Body Image 29, 110–117. doi: 10.1016/j.bodyim.2019.03.002 30921763

[ref22] Bruijn G.-J. de Putte B. van Den. (2012). Exercise promotion: an integration of exercise self-identity, beliefs, intention, and behaviour. Eur. J. Sport Sci., 12, 354–366. doi: 10.1080/17461391.2011.568631

[ref23] De la Torre Cruz M. Lopez S. Ruiz-Ariza A. Martínez-López E. J. (2019). Perceived parental support toward physical activity positively predicts physical self-concept in young adolescents. Educ. Psychol. 39, 941–959. doi: 10.1080/01443410.2019.1620921

[ref24] Deci E. L. Ryan R. M. (2000). The “what” and “why” of goal pursuits: human needs and the self-determination of behavior. Psychol. Inq. 11, 227–268. doi: 10.1207/S15327965PLI1104_01

[ref25] Epskamp S. Fried E. I. (2018). A tutorial on regularized partial correlation networks. Psychol. Methods 23, 617–634. doi: 10.1037/met0000167 29595293

[ref26] Folkman S. (2013). “Stress: appraisal and coping,” in Encyclopedia of behavioral medicine. eds. Gellman M. D. Turner J. R. (New York: Springer), 1913–1915.

[ref27] Fox K. R. Corbin C. B. (1989). The physical self-perception profile: development and preliminary validation. J. Sport Exerc. Psychol. 11, 408–430.

[ref29] Ginis K. A. M. Heisz J. Spence J. C. Clark I. B. Antflick J. Ardern C. I.. (2017). Formulation of evidence-based messages to promote the use of physical activity to prevent and manage Alzheimer’s disease. BMC Public Health 17:209. doi: 10.1186/s12889-017-4090-5, PMID: 28212648 PMC5316179

[ref30] Han Z. Ju H. (2022). The relationship between physical activity and academic engagement among college students-the mediating chain effect of trait mindfulness and self-efficacy. Rev. Psicol. Deporte 31:4.

[ref31] Hausenblas H. A. Fallon E. A. (2006). Exercise and body image: a meta-analysis. Psychol. Health 21, 33–47. doi: 10.1080/14768320500105270

[ref32] Knepp M. M. Yoza J. J. Quandt E. A. (2015). Higher modified Beck depression inventory scores are associated with body, eating, and exercise comparisons but decreased exercise amounts. Percept. Mot. Skills 120, 945–959. doi: 10.2466/15.29.PMS.120v14x8, PMID: 25938448

[ref33] Li B. Tong W. Zhang M. Wang G. Zhang Y. Meng S.. (2022). Epidemiological study of physical activity, negative moods, and their correlations among college students. Int. J. Environ. Res. Public Health 19:748. doi: 10.3390/ijerph191811748, PMID: 36142020 PMC9516961

[ref34] Lin R. Hu X. Chen S. Huang J. (2022). Sports participation and anti-epidemic: empirical evidence on the influence of regular physical activity on the COVID-19 pandemic in mainland China. Int. J. Environ. Res. Public Health 19:10689. doi: 10.3390/ijerph191710689 36078405 PMC9517875

[ref35] Liu Y. Liu S. Liu R. Liu Y. (2023). Leader mindfulness and employee safety behaviors in the workplace: a moderated mediation study. J. Manag. Psychol. 39, 287–303. doi: 10.1108/JMP-03-2022-0128

[ref36] Marsh H. W. Papaioannou A. Theodorakis Y. (2006). Causal ordering of physical self-concept and exercise behavior: reciprocal effects model and the influence of physical education teachers. Health Psychol. 25, 316–328. doi: 10.1037/0278-6133.25.3.316 16719603

[ref37] Meng-long L. Jiao Y. Yu-jia R. (2019). Test of Chinese exercise identity scale in college students. Chin. J. Clin. Psych. 27, 63–66. doi: 10.16128/j.cnki.1005-3611.2019.01.013

[ref38] Mierswa T. Kellmann M. (2017). Differences in low back pain occurrence over a 6-month period between four recovery-stress groups. Work 58, 193–202. doi: 10.3233/WOR-172618 29036865

[ref39] Moreno-Murcia J. A. Hellín P. González-Cutre D. Martínez-Galindo C. (2011). Influence of perceived sport competence and body attractiveness on physical activity and other healthy lifestyle habits in adolescents. Span. J. Psychol. 14, 282–292. doi: 10.5209/rev_SJOP.2011.v14.n1.25 21568185

[ref40] Neumark-Sztainer D. Paxton S. J. Hannan P. J. Haines J. Story M. (2006). Does body satisfaction matter? Five-year longitudinal associations between body satisfaction and health behaviors in adolescent females and males. J. Adolesc. Health 39, 244–251. doi: 10.1016/j.jadohealth.2005.12.001 16857537

[ref41] Ng D. M. Jeffery R. W. (2003). Relationships between perceived stress and health behaviors in a sample of working adults. Health Psychol. 22, 638–642. doi: 10.1037/0278-6133.22.6.638 14640862

[ref42] Nguyen D. T. Wright E. P. Dedding C. Pham T. T. Bunders J. (2019). Low self-esteem and its association with anxiety, depression, and suicidal ideation in Vietnamese secondary school students: a cross-sectional study. Front. Psych. 10:698. doi: 10.3389/fpsyt.2019.00698, PMID: 31611825 PMC6777005

[ref43] Ntoumanis N. Ng J. Y. Y. Prestwich A. Quested E. Hancox J. E. Thøgersen-Ntoumani C.. (2021). A meta-analysis of self-determination theory-informed intervention studies in the health domain: effects on motivation, health behavior, physical, and psychological health. Health Psychol. Rev. 15, 214–244. doi: 10.1080/17437199.2020.1718529 31983293

[ref44] Porter C. D. Kwan M. Y. W. Meca A. Brown D. M. Y. (2024). Exercise identity and physical activity behavior during late adolescence: a four wave cross-lagged panel model. Psychol. Sport Exerc. 73:102641. doi: 10.1016/j.psychsport.2024.102641 38593967

[ref45] Ritchie T. D. Sedikides C. Wildschut T. Arndt J. Gidron Y. (2011). Self-concept clarity mediates the relation between stress and subjective well-being. Self Identity 10, 493–508. doi: 10.1080/15298868.2010.493066

[ref46] Roemmich J. N. Gurgol C. M. Epstein L. H. (2003). Influence of an interpersonal laboratory stressor on youths’ choice to be physically active. Obes. Res. 11, 1080–1087. doi: 10.1038/oby.2003.148 12972678

[ref47] Schneider F. W. Gruman J. A. Coutts L. M. (2005). “Applied social psychology: understanding and addressing social and practical problems,” in Applied social psychology: understanding and addressing social and practical problems (Thousand Oaks, CA: Sage Publications, Inc), 13–449.

[ref48] Schwartz S. J. Klimstra T. A. Luyckx K. Hale W. W. Frijns T. Oosterwegel A.. (2011). Daily dynamics of personal identity and self–concept clarity. Eur. J. Personal. 25, 373–385. doi: 10.1002/per.798

[ref49] Stets J. E. Burke P. J. (2000). Identity theory and social identity theory. Soc. Psychol. Q. 63:224. doi: 10.2307/2695870

[ref50] Stryker S. Serpe R. T. (1982). “Commitment, identity salience, and role behavior: theory and research example,” in Personality, roles, and social behavior. eds. Ickes W. Knowles E. S. (New York: Springer), 199–218.

[ref51] Stults-Kolehmainen M. A. Sinha R. (2014). The effects of stress on physical activity and exercise. Sports Med. 44, 81–121. doi: 10.1007/s40279-013-0090-5, PMID: 24030837 PMC3894304

[ref52] Swami V. Ng S.-K. Barron D. (2016). Translation and psychometric evaluation of a standard Chinese version of the body appreciation Scale-2. Body Image 18, 23–26. doi: 10.1016/j.bodyim.2016.04.005 27236474

[ref53] Swami V. Voracek M. Todd J. Furnham A. Horne G. Tran U. S. (2024). Positive self-beliefs mediate the association between body appreciation and positive mental health. Body Image 48:101685. doi: 10.1016/j.bodyim.2024.101685, PMID: 38382233

[ref54] Tylka T. L. Wood-Barcalow N. L. (2015a). The body appreciation Scale-2: item refinement and psychometric evaluation. Body Image 12, 53–67. doi: 10.1016/j.bodyim.2014.09.006 25462882

[ref55] Tylka T. L. Wood-Barcalow N. L. (2015b). What is and what is not positive body image? Conceptual foundations and construct definition. Body Image 14, 118–129. doi: 10.1016/j.bodyim.2015.04.001 25921657

[ref56] Vani M. Murray R. Sabiston C. (2021). “Body image and physical activity,” in Essentials of exercise and sport psychology: an open access textbook. eds. Zenko Z. Jones L. (Chapel Hill, NC: Society for Transparency, Openness, and Replication in Kinesiology), 150–175.

[ref57] White K. M. Thomas I. Johnston K. L. Hyde M. K. (2008). Predicting attendance at peer-assisted study sessions for statistics: role identity and the theory of planned behavior. J. Soc. Psychol. 148, 473–492. doi: 10.3200/SOCP.148.4.473-492 18807422

[ref58] Zhonglin W. E. N. Baojuan Y. E. (2014). Analyses of mediating effects: the development of methods and models. Adv. Psychol. Sci. 22:731. doi: 10.3724/SP.J.1042.2014.00731

[ref59] Zhou S. Li L. Zhao Y. Cao Y. Peng B. Zheng L. (2021). Physical activity under stress: a perspective of HAPA and individual differences. Int. J. Environ. Res. Public Health 18:12144. doi: 10.3390/ijerph182212144 34831897 PMC8619980

